# Impact of PIPAC-Oxaliplatin on Functional Recovery, Good Days, and Survival in a Refractory Colorectal and Appendiceal Carcinomatosis: Secondary Analysis of the US PIPAC Collaborative Phase 1 Trial

**DOI:** 10.1245/s10434-024-15980-9

**Published:** 2024-09-13

**Authors:** Muhammad Talha Waheed, Nora Ruel, Richard L. Whelan, Marwan Fakih, Yuman Fong, Danielle Deperalta, Amit Merchea, Virginia Sun, Robert Krouse, Thanh H. Dellinger, Mustafa Raoof

**Affiliations:** 1 Department of Surgery, City of Hope National Medical Center, Duarte, CA USA; 2 Computation and Quantitative Medicine, City of Hope National Medical Center, Duarte, CA USA; 3 Department of Surgery, Northwell Health, New York, NY USA; 4 Department of Medical Oncology and Therapeutics Research, City of Hope National Medical Center, Duarte, CA USA; 5 Department of Surgery, Mayo Clinic, Jacksonville, FL USA; 6 Department of Population Sciences, City of Hope National Medical Center, Duarte, CA USA; 7 Department of Surgery, University of Pennsylvania, Philadelphia, PA USA

## Abstract

**Background:**

Pressurized intraperitoneal aerosolized chemotherapy (PIPAC) is a novel, minimally invasive, safe, and repeatable method to treat carcinomatosis. Evidence regarding the clinical benefit (quality of life and survival) of PIPAC compared with that of conventional standard therapy (ST) is lacking.

**Methods:**

This is the secondary analysis of the phase 1 US-PIPAC trial for refractory colorectal and appendiceal carcinomatosis. A PIPAC cohort was compared with a retrospective cohort of consecutive patients receiving ST. The primary outcome was number of good days (number of days alive and out of the hospital). The secondary outcomes were overall survival (OS), progression-free survival (PFS), health-related quality of life (HRQoL), and objective functional recovery (daily step count).

**Results:**

The study included 32 patients (PIPAC, 12; ST, 20) with similar baseline characteristics. Compared with the ST cohort, the PIPAC cohort had lower median inpatient hospital stays (> 24 h) within 6 months (0 vs 1; *p* = 0.015) and 1 year (1 vs 2; *p* = 0.052) and higher median good days at 6 months (181 vs 131 days; *p* = 0.042) and 1 year (323 vs 131 days; *p* = 0.032). There was no worsening of HRQoL after repeated PIPACs. Step counts diminished immediately after PIPAC but returned to baseline within 2–4 weeks. Kaplan–Meier analysis demonstrated a favorable association between receipt of PIPAC and OS (median, 11.3 vs 5.1 months; *p* = 0.036).

**Conclusion:**

Compared with ST, PIPAC was associated with higher number of good days, reduced hospitalization burden, and longer OS without a negative impact on HRQoL with repeated PIPACs. These findings are foundational for evaluation of PIPAC in a randomized clinical trial.

**Supplementary Information:**

The online version contains supplementary material available at 10.1245/s10434-024-15980-9.

Peritoneal metastases (PM) from colorectal cancer (CRC) or appendiceal cancer (AC) have a poor prognosis.^1^ Although surgery (i.e., cytoreduction) is associated with improved survival for carefully selected patients, the majority of patients are not candidates for surgery with or without hyperthermia intraperitoneal chemotherapy (HIPEC).^2^ Systemic chemotherapy is the mainstay of treatment for unresectable CRC-PM and high-grade AC-PM. However, modern systemic chemotherapy has demonstrated limited efficacy. For example, after unresectable CRC-PM treated in a trial of first-line chemotherapy, the median overall survival (OS) was approximately14 months.^3^ In chemotherapy refractory settings, the median survival is less than 6 months.^4^ Progression of disease causes significant detriment to health-related quality of life (HRQoL) necessitating frequent hospitalizations due to bowel obstruction, abdominal pain, symptomatic ascites, biliary or ureteral obstruction, and anorexia, among others.^5^

Pressurized intraperitoneal aerosolized chemotherapy (PIPAC) has emerged as an optimal way to deliver intraperitoneal chemotherapy. It is an attractive option for patients not amenable to resection because it is minimally invasive and repeatable and can be administered concurrently with systemic therapy. Several studies focused on evaluation of PIPAC for PM of CRC origin have established the overall safety and feasibility of this approach.^6–10^ However, comparative data to determine the benefit of PIPAC over standard-of-care systemic therapy are lacking.^11–14^ Furthermore, only limited prospective studies have evaluated the impact of PIPAC on HRQoL.

We previously demonstrated the safety, feasibility, and early efficacy signal of oxaliplatin PIPAC in a chemotherapy refractory cohort of CRC-PM and AC-PM patients.^15^ The current study aimed to compare survival and quality of life (QoL) between patients treated with PIPAC and a matched cohort of patients treated with third-line standard therapy (ST). We hypothesized that by regional control of PM, PIPAC would be associated with a greater number of good days (days alive and out of the hospital)^16^ and preserved QoL.

## Methods

### Study Design and Data Source

This was a retrospective secondary analysis of prospectively collected data from arm 2 of a multicenter phase 1 US-PIPAC trial (NCT04329494) analyzing patients with refractory colorectal and appendiceal carcinomatosis, hereafter referred to as the PIPAC cohort.^17^ The PIPAC cohort data (2020–2022) was compared with the data of an ST cohort. The ST cohort comprised chemotherapy refractory patients treated at the City of Hope National Medical Center (Duarte, CA, USA) for peritoneum-limited CRC and AC with best available ST (including chemotherapy, targeted therapy, immunotherapy, or supportive care).

Patient selection for the ST cohort was performed systematically and involved several steps. Initially, the slicer-dicer, a self-service cohort query tool on the electronic medical record (EMR) was used to identify consecutive patients who had confirmed primary AC or CRC with PM (2019–2022). A list of potential consecutive patients who had peritoneum-limited progression on second-line therapy was generated. Imaging and medical records then were reviewed to identify individuals who would have been otherwise eligible for PIPAC (as per US PIPAC phase 1 inclusion criteria^17^), but were treated a year before the start of the PIPAC trial, were offered the PIPAC trial participation but were not interested in PIPAC, were eligible but opted for other treatments as opposed to the PIPAC trial, or had adhesive disease preventing PIPAC from prior surgery.

To limit selection bias in the ST cohort, we included all consecutive patients who had peritoneum-limited progression with colorectal or appendix primary identified by the EMR and satisfied the inclusion criteria. The study was approved by the institutional review board.

### Patient Inclusion

The patients included in the PIPAC and ST cohorts were adult patients 18 years old or older with histologically confirmed peritoneum-limited invasive appendiceal cancer (AC) or colorectal cancer (CRC) with peritoneal metastasis (PM). Patients were included in the ST cohort if they had progressed on two previous standard-of-care chemotherapeutic regimens. As per the trial protocol, PIPAC cohort patients were allowed to be enrolled if they had progressed on one line of chemotherapy. However, in retrospect, all but two of the PIPAC cohort patients had received two or more prior lines of chemotherapy. Patients were included if they had an Eastern Cooperative Oncology Group (ECOG) performance status (PS) of 2 or lower, no contraindications for laparoscopy, no more than 5 L of ascites, and no candidacy for cytoreduction (CRS) and/or HIPEC.

### Patient Characteristics

We extracted baseline characteristics including age, sex, race/ethnicity, performance status (ECOG), and primary cancer site. Relevant past treatment was also collected including prior number of chemotherapy lines, prior radiation status, and prior CRS with or without HIPEC. Hospitalizations were captured and defined as an in-patient hospital or hospice stay longer than 24 h and calculated as the number of days from the date of admission to the date of discharge.

### Objectives

The primary objective of this analysis was to evaluate and compare the objective QoL (number of good days) at 6 months and 1 year between patients undergoing PIPAC and those treated with ST. Furthermore, the secondary objectives were to determine the survival benefit of PIPAC versus ST by comparing overall survival (OS) and progression-free survival (PFS). For the PIPAC patients, we also assessed HRQoL using the EuroQol five-dimensional descriptive system (EQ-5D-5L) and MD Anderson Symptom Index (MDASI) instruments and estimated functional recovery using daily step count.

### Outcomes

#### Number of Good Days

Good days were defined as days alive and outside the hospital at 6 months and 1 year. This is a validated objective composite end point that takes into account both survival and QoL.^16,18,19^ For the PIPAC cohort, visits related to the PIPAC procedures were not considered as hospitalizations for the purposes of good-days analysis. The patients were not admitted after PIPAC, but kept in extended recovery overnight and discharged the next morning.

##### Survival

For the PIPAC cohort, OS was calculated from the date of the first PIPAC to death. For the ST cohort, OS was calculated from the date of initiation of third-line therapy to death. For the patients starting third-line chemotherapy later than 1 month after progression on second-line therapy and the patients who opted for supportive care after progression on second-line therapy, OS was calculated from the date of progression on second-line therapy plus 1 month. Alive patients in both groups were censored at the date of the last follow-up visit. Progression-free survival was defined as the time from treatment initiation (as defined for OS) to progression or death from any cause.

##### Patient-centered outcomes (HRQoL)

All the PIPAC patients were invited to participate in the analysis by the following self-reported validated HRQoL instruments: EQ-5D-5L Index and MDASI.^20,21^ The EQ-5D-5L instrument consists of five questions regarding mobility, self-care, usual activities, pain and discomfort, and anxiety/depression, with higher scores indicating better health related to the five indicators. Scores were calculated using a geographic (U.S.) time trade-off value set. These scores mirror the overall health assessed by the patients themselves using the Health Today score, which uses a visual analog sliding scale (VAS) ranging from 0 (worst) to 100 (best) possible health. The MDASI instrument uses a 10-point system to account for severity and interference of 13 cancer-related symptoms with activities. These instruments were administered at the time of enrollment (BL), before each cycle of PIPAC treatment (C1, C2, C3; repeated every 6 weeks), and at the time of off-treatment (OffTx).

##### Daily step count

After informed consent, all the PIPAC patients were provided with a commercially available wristband tracking device to remotely quantify and monitor the number of steps as an objective measure of functional recovery.^22,23^ The patients were encouraged to wear the device up to 2 weeks before their first PIPAC to establish their baseline or pre-surgery activity during PIPAC hospitalization, and to continue wearing it up to 4 weeks after each cycle.

### Statistical Analysis

#### Data Reporting

Categorical variables were summarized using counts and percentages, and continuous variables were expressed as mean ± standard deviation (SD) or as median and interquartile range (IQR) based on distribution of the data. Univariate analyses for group differences in the baseline characteristics were performed using two-sided Pearson’s chi-square test or two-sided Fisher’s exact test where appropriate. For a priori directional hypotheses, univariate analyses for group differences in outcome characteristics were performed using one-sided Fisher’s exact test for categorical variables and the Mann–Whitney *U* test with one-sided *p* values for continuous variables. Patients with complete follow-up data within 6 months or 1-year were used to report outcome differences for the number of good days. Differences in OS and PFS on bivariate levels were visualized using Kaplan–Meier (KM) curves and assessed using log-rank testing. Differences in EQ-5D-5L and MDASI measures were evaluated for patients who responded to the surveys at two or more time points (*n* = 8) using one-way analysis of variance (ANOVA) to test for differences in PIPAC cycles. The results reported were stratified by stable disease (SD; *n* = 6) versus progressive disease (PD; *n* = 6) according to best overall response evaluated by Response Evaluation Criteria in Solid Tumors (RECIST) version 1.1.

For the step-count data, median daily steps relative to each PIPAC date were plotted in series to help describe both the pre-surgical or baseline steps and the time to (steps) recovery after each PIPAC treatment. A smoothing function was applied to allow for improved visualization, and results were stratified by SD (*n* = 2) and PD (*n* = 4). The remaining six patients in the PIPAC cohort did not participate in the daily step tracking. The median steps recorded during time intervals both before the first PIPAC and during the recovery weeks after surgery were compared between the SD and PD patients using the non-parametric Wilcoxon rank sum-test. The median value for each patient was used during each interval to represent the number of steps.

#### Software

Data were analyzed using STATA (version 16.0) software and SAS^®^9.4. Statistical significance was defined as a *p* value lower than 0.05.

## Results

The study included 32 patients (PIPAC cohort, 12; ST cohort, 20) for the analysis. The clinicopathologic characteristics of the full cohort stratified by cohort type are summarized in Table 1.

**Table 1:** Baseline characteristics of ST and PIPAC cohorts

Characteristic	ST cohort (*n* = 20) *n* (%)	PIPAC (*n* = 12) *n* (%)	*p* Value
Median age: years (IQR)	64 (52–71)	59 (45–62)	0.09
Sex			
Male	10 (50.0)	7 (58.3)	0.7
Female	10 (50.0)	5 (41.7)	
Race/ethnicity			
Non-Hispanic white	10 (50.0)	8 (66.7)	0.6
Black	2 (10.0)	0 (0)	
Hispanic	1 (5.0)	1 (8.3)	
Asian/Pacific Islander	5 (25.0)	2 (16.6)	
Undisclosed/unknown	2 (10.0)	1 (8.3)	
Performance status (ECOG)			
0	7 (35.0)	8 (66.7)	0.1
1	13 (65.0)	4 (33.3)	
Primary tumor site			
Appendiceal	3 (15.0)	4 (33.3)	0.5
Colon	14 (70.0)	6 (50)	
Rectal/rectosigmoid	3 (15.0)	2 (16.7)	
Prior lines of chemotherapy: median (IQR)	2 (2–2)	2 (1.3–3.8)	0.4
Prior radiation			
No	18 (90.0)	10 (83.3)	0.6
Yes	2 (10.0)	2 (16.7)	
Prior cytoreduction/HIPEC			
No	14 (70.0)	8 (66.7)	1.0
Yes	6 (30.0)	4 (33.3)	
No. of PIPAC cycles completed			
1	–	4 (33.3)	–
2	–	2 (16.7)	
≥ 3	–	6 (50.0)	

*ST* standard therapy; *PIPAC* pressurized intraperitoneal aerosolized chemotherapy; *IQR* interquartile range; *ECOG* Eastern Cooperative Oncology Group; *HIPEC* hyperthermic intraperitoneal chemotherapy

### Baseline Characteristics

Baseline characteristics were similar between the two treatment cohorts (Table 1). The PIPAC cohort had a higher number of appendix cancer patients (*n* = 4, 33 %) than the ST cohort (*n* = 3, 15 %), although the difference was not significant. Previous CRS ± HIPEC had been performed for 4 (33.3 %) of the 12 PIPAC cohort patients and 6 (30 %) of the 20 ST cohort patients. The type of CRS ± HIPEC for both cohorts is detailed in Table S1. The ST cohort received the following therapies: continued/re-challenged first- and second-line therapy (oxaliplatin- or irinotecan-based [45 %, 9/20]), third-line therapy (trifluridine/tipiracil or regorafinib [30 %, 6/20]), clinical trial/off-label therapy [15 %, 3/20]), and best supportive care alone [10 %, 2/20]). The patients in the PIPAC cohort did not receive any systemic chemotherapy as part of the trial except for a sensitizing dose of 5-fluorouracil 24 h before the PIPAC procedure. All the patients except two in the PIPAC cohort had received two or more prior lines of irinotecan- and platinum-based chemotherapy. The two patients had foregone second-line therapy to enroll in the trial due to intolerance or concerns for toxicity.

### Hospitalizations and Number of Good Days

**Fig. 1:**
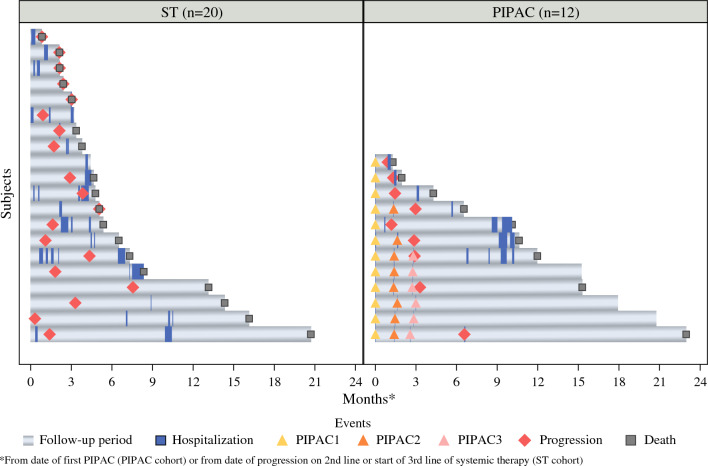
Swimmers’ plot of the two cohorts (ST, standard therapy; *n* = 32)

Follow-up data beyond 6 months was available for 30 patients (PIPAC cohort, 12; ST cohort, 18). In contrast to the ST cohort, the PIPAC cohort had a lower median number of inpatient hospital stays longer than 24 h within 6 months (0 [IQR, 0–1] vs 1 [IQR, 0–2]; *p* = 0.02) and within 1 year (1 [IQR, 0–1.75] vs 2 [IQR, 1–2.25]; *p* = 0.05). Similarly, compared with the ST patients, the patients undergoing PIPAC had a higher median number of good days at 6 months (181 days [IQR, 151–184 days] vs 131 days [IQR, 90–180 days]; *p* = 0.04) and at 1 year (323 days [IQR, 160–365 days] vs 131 days [IQR, 90–227 days]; *p* = 0.03) (Table 2). Compared with the ST cohort, the PIPAC cohort also had a higher median percentage of un-hospitalized days alive at 6 and 12 months, corrected for overall survival (Table S2). Figure 1 and Table S2 show the treatment course of each patient in the two cohorts and highlight the duration of each hospital stay and the outcome for each patient.

**Table 2:** Comparison of hospital stays and good days between the ST and PIPAC cohorts

Characteristic	ST cohort (*n* = 20)^a^	PIPAC cohort (*n* = 12)	*p* value
6-Month hospital stays: *n* (%)			
No	5 (27.8)	7 (58.3)	0.098
Yes	13 (72.2)	5 (41.7)	
1-Year hospital stays: *n* (%)			
No	2 (11.1)	4 (33.8)	0.2
Yes	16 (88.9)	8 (66.7)	
6-Month hospital stays: median (IQR)	1 (0–2)	0 (0–1)	0.015
1-Year hospital stays: median (IQR)	2 (1–2.25)	1 (0–1.75)	0.052
Good days in 6 months: median (IQR)	131 (90–180)	181 (151–184)	0.042
Good days in 1-year: median (IQR)	131 (90–227)	323 (160–365)	0.032
Good days in 6 months: mean ± SD	127 ± 53	154 ± 54	0.042
Good days in 1 year: mean ± SD	170 ± 115	262 ± 128	0.032

*ST* standard therapy; *PIPAC* pressurized intraperitoneal aerosolized chemotherapy; *IQR* interquartile range; *SD* standard deviation

^a^Two patients were excluded from this analysis because they did not have at least 6 months of follow-up evaluation

### Survival Outcomes

**Fig. 2:**
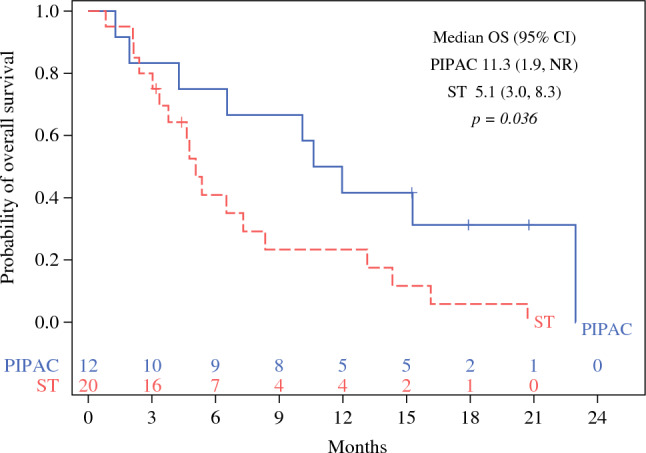
Kaplan-Meier analysis of overall survival (OS) stratified by cohort type (ST, standard therapy; *n* = 32)

**Fig. 3:**
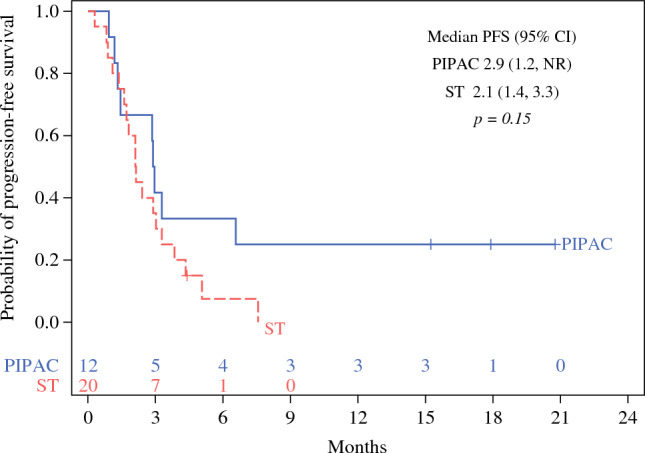
Kaplan-Meier analysis of progression-free survival (PFS) stratified by cohort type (ST, standard therapy; *n* = 32)

The median follow-up time was 20.8 months (95 % confidence interval [CI], 15.2–NR) for the entire cohort. The median OS for the PIPAC cohort was significantly longer (11.3 months; 95 % CI, 1.9–NR) than for the ST cohort (5.1 months; 95 % CI, 3.0–8.3 months) (*p* = 0.036, log-rank; Fig. 2). The PFS was longer for the PIPAC cohort (2.9 months; 95 % CI, 1.2–NR) than for the ST cohort (2.1 months; 95 % CI, 1.4–3.3 months), but this difference was not statistically significant (*p* = 0.1, log-rank; Fig. 3).

### Patient-Reported Outcomes for the PIPAC Cohort

Granular patient-centered outcomes were available for the PIPAC cohort. All the patients except one completed the baseline surveys. The surveys were completed at C1 by nine patients, at C2 by seven patients, and at C3 by three patients. Five patients completed off-treatment exit surveys.

**Fig. 4:**
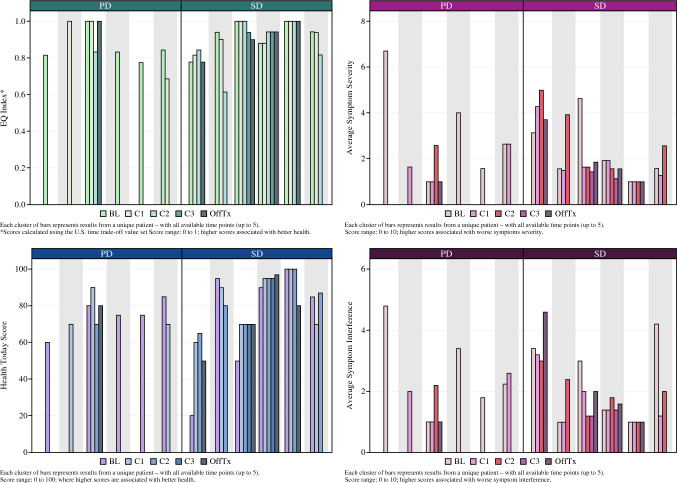
EQ-5D-5L and MDASI for SD versus PD patients (SD, stable disease; PD, progressive disease; *n* = 12). EQ-5D-5L, EuroQol five-dimensional descriptive system; MDASI, MD Anderson Symptom Index

Figure 4 presents HRQoL outcomes stratified by PD (*n* = 6) and SD (*n* = 6). The number of QoL questionnaires completed varied (range, 1–5) based on the number of PIPACs completed and patient adherence, which was moderate; The questionnaires were completed by 5 (42 %) of the 12 PIPAC patients at all the treatment cycles as well as at off-treatment. In addition, 4 (33 %) of the 12 patients completed the surveys at all but one of the prescribed time points. Overall, the QoL scores did not worsen with repeated PIPAC cycles or subsequent time points (*p* > 0.6, one-way ANOVA, all QoL metrics; Fig. S1).

**Fig. 5:**
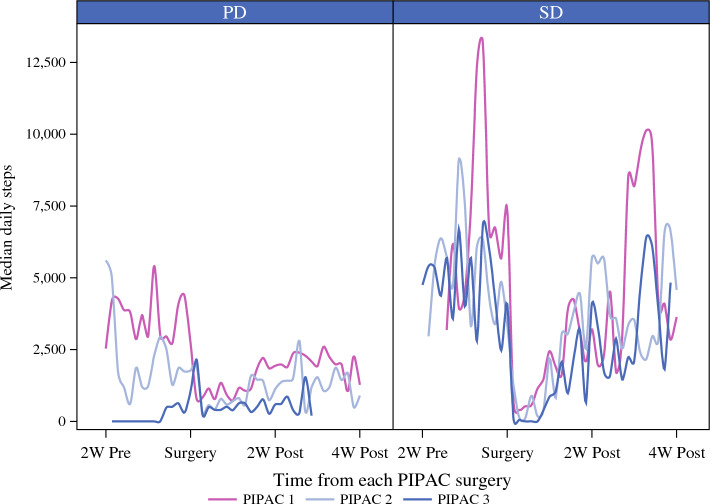
Step-count data for SD vs PD patients (SD, stable disease; PD, progressive disease; *n* = 6)

Six patients participated in step-count monitoring. Step-count data with respect to each PIPAC cycle is plotted in Fig. 5 stratified by PD (*n* = 2) and SD (*n* = 4). The figure demonstrates a decline in step count after PIPAC, with a gradual return to baseline in 2 to 4 weeks. Furthermore, compared with the SD patients, the PD patients showed a relatively lower median baseline step count and failure to recover baseline median step count within 4 weeks after surgery. Individual step-count data for all six patients are plotted in Fig. S2.

## Discussion

Unresectable CRC and AC metastases to the peritoneum are a complex clinical challenge with high morbidity, limited treatment options, and a poor prognosis. Although PIPAC has emerged as a novel therapeutic option, data comparing the benefit of PIPAC over ST are lacking. This is the first study to compare the efficacy of PIPAC with that of ST for patients with chemorefractory CRC-PM or AC-PM. The findings demonstrated that for patients with chemorefractory peritoneum-limited unresectable peritoneal metastases from invasive AC or CRC, PIPAC may be associated with better QoL (2.5-fold higher number of median good days at 1 year) and OS (59 % reduction in risk of death) than ST. Additionally, compared with the ST cohort, the PIPAC cohort had a lower burden of hospitalization during the course of 1 year after the start of treatment. Subjective QoL measured using EQ-5D-5L, Health Today scores, and MDASI showed preserved QoL after repeated PIPAC cycles. Finally, step-count data demonstrated a decline in activity immediately after PIPAC, which returned to baseline after 2 to 4 weeks, providing an objective benchmark for functional recovery.

Most patients with cancer nearing the end of life want to stay at home or at least be cared for at home.^24^ “Good days” is a composite outcome that combines survival and QoL. No previous reports describe using “good days” as an outcome measure for PIPAC, but it is a validated objective composite end point with demonstrated utility in a randomized trial of malignant bowel obstruction (S1316).^16^ The increase in number of good days for patients undergoing PIPAC (compared with ST) suggests a potentially meaningful clinical benefit of PIPAC in a chemorefractory setting. These observations also provide a rationale for the use of good days as a primary end point in trials of refractory peritoneal metastases.

To perform a granular assessment of HRQoL, we used two validated instruments: EQ-5D-5L and MDASI. Overall, we found no decline in HRQoL with repeated PIPAC treatment cycles. In contrast, Lurvink.et al.^6^ reported reversible worsening of patient-related outcomes using EQ-5D-5L in the first week after PIPAC cycle 1. The discordance can be explained by the differences in the timing of the surveys between the two studies. Several other studies have evaluated patient-centered outcomes after PIPAC for PM from various primary cancers including colorectal cancer, but have been more focused on ovarian, gastric, peritoneal mesothelioma, endometrial, and breast primary cancers.^6,9,25–36^ These studies used various sets of surveys and questionnaires including EQ-5D-5L, European Organization for Research and Treatment of Cancer (EORTC) QLQ-30, and Short Form 36 (SF-36; Medical Outcome Trust, Health Assessment Laboratories and QualityMetric, Lincoln, RI, USA). Our observations are in agreement with most of the prior studies that demonstrated no detriment in QoL scores with repeated PIPAC treatment cycles.^25–30,37^

In palliative studies, survey-based assessment of patient-reported outcomes can be confounded by significant attrition as the disease progresses. Functional assessment of patient functional recovery has previously been reported in prospective surgical cohorts, but not in chemotherapy trials. This is the first study to assess “daily number of steps” after PIPAC as a unique outcome of functional recovery. The results demonstrated that step counts decrease after PIPAC but return to near baseline 2 to 4 weeks after PIPAC administration, suggesting that PIPAC may have a limited but reversible impact on patient QoL. Unlike the QoL scores, which did not differ between the PD and SD patients, the step counts were higher at baseline for the SD patients than for the PD patients. Similarly, the SD patients demonstrated a more robust recovery to normal step counts after PIPAC than the PD patients. These observations raise the possibility that baseline functional activity, once validated in future studies, may be used to select or stratify patients in PM clinical trials.

The comparative analysis of survival between the two comparable cohorts also suggested a potential benefit of PIPAC. The median OS (5.1 months) and PFS (2.1 months) of the ST cohort were comparable with those in published trials of two major third-line systemic therapies for CRC with PM, namely, regorafenib (OS, 6.4 months; PFS, 1.9 months) and trifluridine/tipiracil (OS, 7.1 months; PFS, 2 months).^4,38^ The median OS (11.3 months) and PFS (2.9 months) of the PIPAC cohort compare favorably with the results from both our ST cohort and prior trials of third-line systemic chemotherapy. A shorter survival was reported by the PIPAX-OX trial (OS, 4.1 months; PFS, 1.5 months), but this decrease might be explained by the inclusion of various different primary cancers, which reflect different disease biologies. Moreover, 3 of the 16 patients in PIPAC-OX trial had prior extraperitoneal disease, and none of the patients were able to complete all three PIPAC cycles.^13^ Analysis of the data from two prospective trials of CRC-PM (PIPAC-OPC1 and PIPAC OPC-2) reported a longer median OS (20.5 months) from the time of the first PIPAC treatment cycle.^10^ However, the results are not comparable with those of the current study because the PIPAC-OPC cohorts were more heterogeneous, with a lack of strict inclusion criteria regarding prior systemic therapy. For example, although most of the patients (91 %, 22/24) had received palliative chemotherapy before enrolling in the PIPAC trial, the majority (63 %) had received only first-line chemotherapy with or without progression. In addition, one third of the patients had an extremely low disease burden (peritoneal carcinomatosis index [PCI], < 2.6). The longer survival of the PIPAC-OPC cohorts may be explained by these differences in inclusion criteria between the two studies.

This study had several limitations. Although PIPAC was associated with improved OS, this result does not prove the superiority of PIPAC over third-line systemic chemotherapy for unresectable PM. This result can be interpreted only as hypothesis-generating to evaluate PIPAC in a future randomized trial. The study also was limited by its small sample and its retrospective design. Nevertheless, it built on the results of the first in the U.S phase 1 PIPAC trial and provided a comparison group for comparing outcomes. Although the two groups had similar baseline characteristics for measured variables, we cannot rule out the possibility that the differences in outcomes could have been related to unmeasured confounders. Functional recovery and QoL analyses are limited due to small samples. A larger sample and improved participation would paint a clearer picture of the relevant trends. Nonetheless, these results provide a snapshot of QoL and functional recovery, which are important to consider and incorporate in future PIPAC studies.

In conclusion, given its safety, lack of negative impact on QoL, and improved efficacy signal, PIPAC needs to be investigated further as a treatment option for refractory, isolated peritoneal metastasis of CRC or AC origin in a randomized clinical trial against standard-of-care systemic therapy. These early promising results provide foundational evidence for the use of good days at 1 year as a primary end point for a future PIPAC trial in chemotherapy refractory setting. Finally, the results demonstrate the feasibility of integrating step-count data in prospective clinical trials of intra-peritoneal therapy as an objective surrogate for functional recovery.

## Supplementary Information

Below is the link to the electronic supplementary material.

Supplementary file1 (DOCX 324 kb)
