# Supplementary material for: Impact of PIPAC-Oxaliplatin on Functional Recovery, Good Days, and Survival in a Refractory Colorectal and Appendiceal Carcinomatosis: Secondary Analysis of the US PIPAC Collaborative Phase 1 Trial
Source: Ann Surg Oncol. 2024 Sep 13;31(12):7998–8007. doi: 10.1245/s10434-024-15980-9 (PMC11467104; doi:10.1245/s10434-024-15980-9)

**Impact of PIPAC-Oxaliplatin on Functional Recovery, Good Days, and Survival in Refractory Colorectal and Appendiceal Carcinomatosis: Secondary analysis of the US PIPAC Collaborative Phase 1 trial**

Muhammad Talha Waheed MD^1^, Nora Ruel M.A.^2^, Richard L. Whelan MD^3^, Marwan Fakih MD^4^, Yuman Fong MD^1^, Danielle Deperalta MD^3^, Amit Merchea MD^5^, Virginia Sun PhD^6^, Robert Krouse MD^7^, Thanh H. Dellinger MD^1^, Mustafa Raoof MD^1^

**Supplemental information:**

**Supplemental Table 1**. Type of past CRS +/- HIPEC for ST and PIPAC Cohort.

| **PIPAC Cohort (N=4/12)** | | | |
| --- | --- | --- | --- |
| *Patient* | *Organ resections* | *HIPEC* | *Histology* |
| 1 | Subtotal colectomy, partial gastrectomy, splenectomy, omentectomy, peritonectomy | Yes | Appendix |
| 2 | Total abdominal hysterectomy and bilateral salpingo-oophorectomy (TAHBSO) | No | Colon |
| 3 | Oophorectomy | No | Appendix |
| 4 | Bilateral salpingo-oophorectomy (BSO) | Yes | Colon |
| **ST Cohort (N=6/20)** | | | |
| *Patient* | *Organ resections* | *HIPEC* |  |
| 1 | Cholecystectomy, partial splenectomy, Right hemicolectomy and terminal ileum, partial small bowel resection, total abdominal hysterectomy, omentectomy, peritonectomy, abdominal wall resection | No | Colon |
| 2 | Small bowel resection x2, palliative total abdomino-perineal pelvic exenteration, hernia excision | No | Rectal/ Rectosigmoid |
| 3 | Appendectomy, low anterior resection, omentectomy, peritonectomy | Yes | Colon |
| 4 | Cholecystectomy, small bowel resection, TAHBSO, omentectomy, peritonectomy | No | Colon |
| 5 | Cholecystectomy, small bowel resection, extended right hemicolectomy, low anterior resection, TAHBSO, peritonectomy, omentectomy, abdominal wall resection | Yes | Colon |
| 6 | Cholecystectomy, low anterior resection, partial cystectomy, omentectomy, peritonectomy | No | Appendix |

**Supplemental Table 2.** Percentage of days alive & outside of the hospital at 6 and 12 months, based on overall survival [(Unhospitalized days)/(Days alive)].

|  | Median % (IQR) | | p-value |
| --- | --- | --- | --- |
| Time-point | ST (n=20) | PIPAC (n=12) |  |
| 6 months | 96.2 (IQR: 86.8 – 99.2) | 100 (IQR: 97.3 – 100) | 0.018 |
| 12 months | 95.1 (IQR: 86.0 – 97.9) | 97.3 (IQR: 91.4 – 100) | 0.066 |

**Supplemental Table 3.** Detailed hospitalization and survival data for ST and PIPAC cohorts with primary disease site.

| **Cohort** | **ID** | **Disease Site** | **No. of PIPACs** | **Total**  **Hospitalization**  **days** | **PFS**  **months** | **OS**  **months** |
| --- | --- | --- | --- | --- | --- | --- |
| ST | 1 | Colon |  | 20 | 1.4 | 20.7 |
| ST | 2 | Colon |  | 8 | 0.3 | 16.1 |
| ST | 3 | Colon |  | 1 | 3.3 | 14.3 |
| ST | 4 | Colon |  | 0 | 7.6 | 13.1 |
| ST | 5 | Colon |  | 27 | 1.8 | 8.3 |
| ST | 6 | Appendix |  | 34 | 4.3 | 7.3 |
| ST | 7 | Rectum/Rectosigmoid |  | 3 | 1.1 | 6.5 |
| ST | 8 | Colon |  | 23 | 1.6 | 5.4 |
| ST | 9 | Colon |  | 6 | 5.1 | 5.1 |
| ST | 10 | Colon |  | 23 | 3.8 | 4.8 |
| ST | 11 | Rectum/Rectosigmoid |  | 14 | 2.9 | 4.6 |
| ST | 12 | Colon |  | 5 | 4.4 | 4.4 |
| ST | 13 | Colon |  | 5 | 1.7 | 3.8 |
| ST | 14 | Appendix |  | 2 | 2.1 | 3.4 |
| ST | 15 | Colon |  | 15 | 0.9 | 3.2 |
| ST | 16 | Colon |  | 2 | 3.0 | 3.0 |
| ST | 17 | Colon |  | 0 | 2.4 | 2.4 |
| ST | 18 | Colon |  | 9 | 2.1 | 2.1 |
| ST | 19 | Appendix |  | 8 | 2.1 | 2.1 |
| ST | 20 | Rectum/Rectosigmoid |  | 9 | 0.8 | 0.8 |
| PIPAC | 1 | Rectum/Rectosigmoid | 3 | 2 | 6.6 | 23.0 |
| PIPAC | 2 | Colon | 3 | 0 | 20.8 | 20.8 |
| PIPAC | 3 | Appendix | 3 | 0 | 17.9 | 17.9 |
| PIPAC | 4 | Rectum/Rectosigmoid | 3 | 0 | 3.3 | 15.3 |
| PIPAC | 5 | Colon | 3 | 0 | 15.2 | 15.2 |
| PIPAC | 6 | Appendix | 3 | 23 | 2.9 | 12.0 |
| PIPAC | 7 | Colon | 2 | 28 | 2.9 | 10.6 |
| PIPAC | 8 | Colon | 1 | 36 | 1.2 | 10.1 |
| PIPAC | 9 | Colon | 2 | 3 | 3.0 | 6.5 |
| PIPAC | 10 | Appendix | 1 | 5 | 1.4 | 4.3 |
| PIPAC | 11 | Appendix | 1 | 5 | 1.3 | 1.9 |
| PIPAC | 12 | Colon | 1 | 6 | 0.9 | 1.3 |

**Supplemental figure 1**. Anova to test overall difference of measures over time (where Cycle1= 1, Cycle2 = 2, Cycle3 = 3 and off-treatment=4) for patients who responded to the surveys at ≥ 2 time-points (N=8).

**
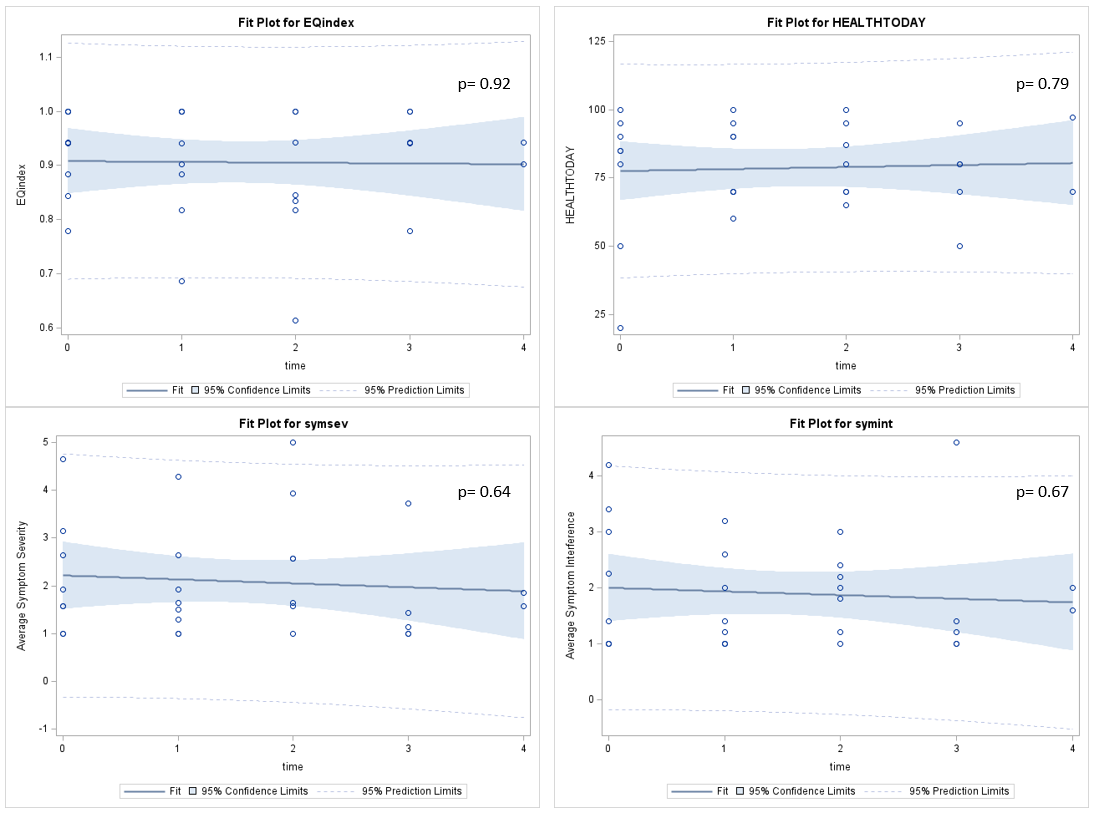
**

**Supplemental Figure 2.** Individual step-count data for all six patients that participated in step-count monitoring.


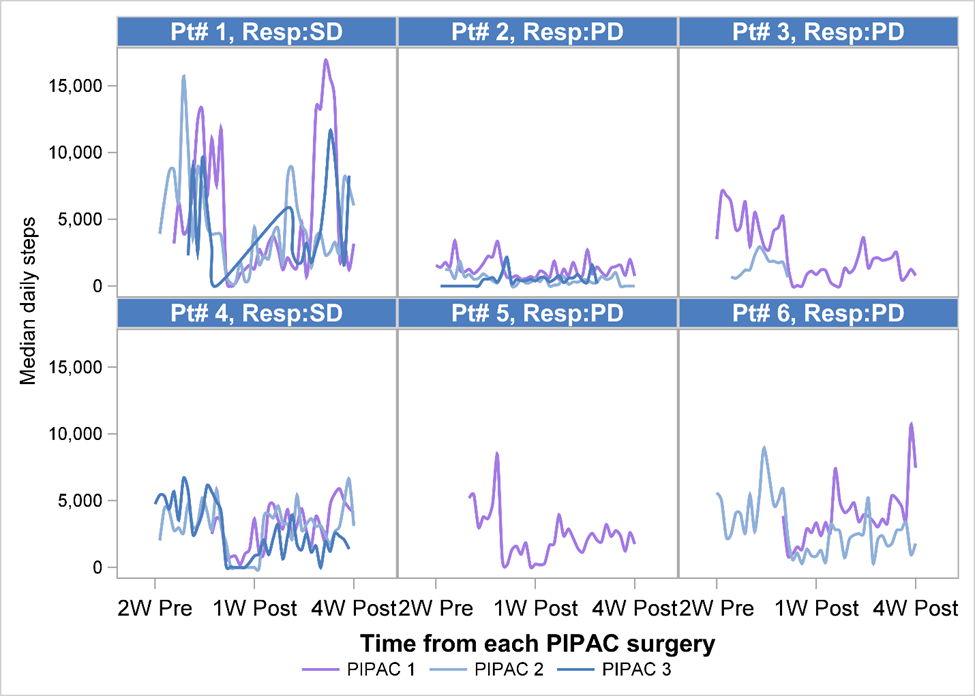

Supplement: Supplementary file 1 — Supplementary file1 (DOCX 324 kb) [file 10434_2024_15980_MOESM1_ESM.docx]
